# Next-Generation Approaches in Sports Medicine: The Role of Genetics, Omics, and Digital Health in Optimizing Athlete Performance and Longevity—A Narrative Review

**DOI:** 10.3390/life15071023

**Published:** 2025-06-27

**Authors:** Alen Juginović, Adrijana Kekić, Ivan Aranza, Valentina Biloš, Mirko Armanda

**Affiliations:** 1 Department of Neurobiology, Harvard Medical School, 220 Longwood Avenue, Boston, MA 02115, USA; 2 Department of Pharmacy, Mayo Clinic Arizona, 5777 E Mayo Blvd, Phoenix, AZ 85054, USA; 3 Department of Cardiology, University Hospital Split, Spinčićeva 1, 21000 Split, Croatia; 4 Institute of Emergency Medicine of the Zagreb County, Radićev Odvojak 58, 10410 Velika Gorica, Croatia; 5 School of Medicine, University of Split, Šoltanska 2, 21000 Split, Croatia

**Keywords:** precision sports medicine, genomics, pharmacogenomics, multi-omics, digital health

## Abstract

This review aims to provide a comprehensive framework for implementing precision sports medicine, integrating genetics, pharmacogenomics, digital health solutions, and multi-omics data. Literature review was conducted using MEDLINE, EMBASE, Web of Science, and Cochrane Library databases (January 2018–April 2024), focusing on precision medicine applications in sports medicine, utilizing key terms including “precision medicine”, “sports medicine”, “genetics”, and “multi-omics”, with forward and backward citation tracking. The review identified key gene variants affecting athletic performance: endurance (*AMPD1*, *PPARGC1A*), power (*ACTN3*, *NOS3*), strength (*PPARG*), and injury susceptibility (*COL5A1*, *MMP3*), while also examining inherited conditions like cardiomyopathies (*MYH7*, *MYBPC3*). Pharmacogenomic guidelines were established for optimizing common sports medications, including NSAIDs (*CYP2C9*), opioids (*CYP2D6*), and cardiovascular drugs (*SLCO1B1*, *CYP2C19*). Digital health technologies, including wearables and predictive analytics, showed potential for enhanced athlete monitoring and injury prevention, while multi-omics approaches integrated various molecular data to understand exercise capacity and injury predisposition, enabling personalized assessments, training regimens, and therapeutic interventions based on individual biomolecular profiles. This review provides sports medicine professionals with a framework to deliver personalized care tailored to each athlete’s unique profile, promising optimized performance, reduced injury risks, and improved recovery outcomes.

## 1. Introduction

Sports medicine has traditionally applied evidence-based practices derived from research on athletic populations as a whole. However, an increasing body of research is highlighting the influential role of individual factors like genetics, physiology, and biomechanics in determining an athlete’s performance capabilities, injury risks, and responses to interventions [1,2]. This growing appreciation for individual variability has given rise to precision sports medicine—an emerging field that aims to provide personalized assessments, training sessions, and treatments tailored to each athlete’s unique characteristics [3]. Rather than relying solely on generalized population data, precision approaches leverage next-generation technologies, genetics, and multi-omics data to develop customized athlete management strategies. The overarching goal is to help athletes perform at their top level while ensuring sustainable top quality medical care.

This shift toward personalization promises to improve multiple aspects of sports medicine, from disease prevention and injury mitigation to optimized training regimens and maximizing athletic capabilities [4,5,6]. The potential impact could extend beyond elite competitions, offering benefits for recreational athletes, active individuals, and society through enhanced health, longevity, and quality of life. While personalization might not completely revolutionize the field of sports medicine as we know it today, it has the potential to make incremental, yet significant advancements for the health of athletes, where every percent of improvement in terms of medical care is welcome, benefiting both performance and career longevity.

At the core of personalized sports medicine lies genetics and its influence on an individual’s physical capabilities, injury vulnerabilities, and recuperation capacity [3]. Genome-wide association studies have uncovered genetic variants linked to traits important for sports excellence. For example, variants in the *ACTN3* gene modulate muscle fiber composition and sprint performance [7], while polymorphisms in *COL5A1* have been implicated in predisposition to anterior cruciate ligament injuries, which are among the ones with the longest recovery time and could even be career-ending for some [8]. By integrating an athlete’s genetic profile into clinical and on-field decision making, practitioners can tailor the intensity, timing, and content of training programs, implement targeted injury prevention strategies, and potentially predict the likelihood of an individual’s propensity for specific sports-related injuries more accurately.

Complementing genetics is pharmacogenomics (PGx), which investigates how an individual’s genetic makeup influences their response to therapeutic interventions [9]. This is especially significant in athletes, where every day of being off the field is a day lost in terms of career advancement. Studies have shown that genetic variants can significantly alter the pharmacokinetic and pharmacodynamic properties of common sports medications, such as anti-inflammatory drugs like ibuprofen (impacted by CYP2C9 enzyme variants [10]. Using information about the athlete’s genetic profile, where the sample is usually taken from a simple cheek swab, sports physicians can optimize medication dosages, minimize adverse reactions, and enhance therapeutic effectiveness, facilitating faster and more efficient recovery and safer return to play [10,11].

The recent rapid proliferation of digital health technologies is transforming athlete monitoring, coaching, and medical care even today. Considering how quickly technology evolves, one could easily imagine that digital health could stay a core part of every sports physicians’ playbook in the future. Wearable devices, mobile health apps, and telemedicine platforms are just some of the examples that have ushered in real-time physiological tracking, personalized training sessions, and remote clinical services [11,12]. Elite teams are integrating multi-sensor data streams into centralized athlete management systems, providing in-depth insight into individual readiness, performance capabilities, and areas requiring intervention. These digital health technologies are driving a shift towards proactive, data-driven, and highly personalized approaches to athlete management and care delivery.

A relatively new field in sports medicine is multi-omics, which integrates comprehensive datasets from various “omics” disciplines (e.g., proteomics, metabolomics), and provides a multi-dimensional view of an individual’s molecular landscape [5]. While it is still emerging in the context of sports, early studies have revealed distinct multi-omic signatures in elite athletes, offering novel insights into the molecular mechanisms underlying athletic performance [13,14]. By slowly adopting the novel multi-omics approaches, sports medicine professionals can better understand the interplay between genetic factors, environmental influences, and lifestyle choices that shape an athlete’s physiology and potential. This enables the development of personalized, multi-pronged interventions that simultaneously target multiple biological processes. For example, an athlete’s multi-omic profile could inform tailored dietary recommendations, customized training loads, and targeted supplementation to enhance energy metabolism, muscle recovery, and injury resilience.

While precision sports medicine principles can be applied across sexes, it is important to recognize potential male-female differences influencing implementation. Studies indicate sex-based variations in muscle physiology, biomechanics, hormones, and injury patterns that could impact training, recovery, and injury risk [15,16]. The influence of certain gene variants on traits like muscle performance may be modulated by sex hormones [17], and PGx data reveals sex differences in drug metabolism for some medications commonly used in sports medicine [18].

While the potential of precision sports medicine is vast, the practical implementation of novel technologies and tests in clinical settings remains understandably challenging, both from a logistical side, as well as many professionals needing additional education in these novel fields. This paper aims to bridge this gap by providing sports medicine professionals with a thorough review of the latest advancements, actionable strategies, and benefits for integrating genetics, PGx, digital health solutions, and multi-omics data into daily practice. Our goal is to raise awareness and to equip practitioners with the knowledge, tools, and guidance necessary to deliver the next-generation precision sports medicine tailored to each athlete’s unique genetic makeup, physiological profile, and performance goals. By translating scientific findings into clinical utility, we aim to help sports physicians in promoting long-term athlete well-being and sustained competitive excellence.

## 2. Methods

### 2.1. Literature Search and Review Process

We conducted a comprehensive narrative review to synthesize current knowledge and emerging trends in precision sports medicine, focusing on genomics, PGx, multi-omics, and digital health technologies. Our search strategy involved a thorough exploration of the literature using electronic databases including MEDLINE (via PubMed), EMBASE, and Web of Science. We focused on articles published between January 2018 and April 2024 to capture the most recent developments in this rapidly evolving field.

Search terms included combinations of keywords related to “precision medicine”, “sports medicine”, “genomics”, “pharmacogenomics”, “digital health”, and “multi-omics.” We also performed forward and backward citation tracking of key articles to ensure comprehensive coverage of the topic.

### 2.2. Selection Criteria and Data Extraction

We included peer-reviewed studies in English that examined precision medicine approaches in sports, encompassing human subject research, animal models, in vitro experiments, and other review articles. We excluded case reports, conference abstracts, and opinion pieces. The selection of articles was based on their relevance to the field of precision sports medicine and their potential to inform clinical practice or future research directions.

From the selected studies, we extracted information on study design, precision medicine approach, key findings, and implications for sports medicine. For human studies, we noted population characteristics and sport type where relevant. For animal and in vitro studies, we considered their translational potential to human athletes.

### 2.3. Data Synthesis and Framework Development

Given the diverse nature of the included studies, we performed a qualitative synthesis rather than a meta-analysis. We organized our findings into four main domains: genomics, PGx, digital health technologies, and multi-omics approaches in sports medicine. Within each domain, we summarized the current state of knowledge, identified emerging trends, and highlighted knowledge gaps. Based on our synthesis, we developed a framework for implementing precision sports medicine in clinical practice. This framework was iteratively refined through discussions among our multidisciplinary team.

### 2.4. Limitations

As a narrative review, our study is subject to potential biases in article selection and interpretation. While we aimed for a comprehensive overview, we acknowledge that some relevant studies may have been inadvertently omitted. Furthermore, the rapidly evolving nature of precision medicine means that new developments may have occurred since the completion of our review.

## 3. Results

### 3.1. Genetics

Research has unveiled the significant impact of genetic factors on athletic performance and health [19,20]. Genetic factors, for example, account for a large 66% of the variability in athletic ability and status [21], underscoring the importance of identifying specific genetic variants that determine performance potential, injury susceptibility, and sports-related medical risks.

Several genetic variants have been found to influence athletic endurance, power, and strength domains. Endurance-related alleles (e.g., *AMPD1* rs17602729 C, *PPARGC1A* rs8192678 G) are connected to factors like muscle fiber type, hemoglobin mass, and maximal oxygen consumption [19,20,22,23]. Power performance is associated with alleles (e.g., *ACTN3* rs1815739 C, *NOS3* rs2070744 T) influencing testosterone levels, muscle characteristics, and reaction time [24,25]. Strength is shaped by alleles (e.g., *ACTN3* rs1815739 C, *PPARG* rs1801282 G) modulating muscle hypertrophy, fiber type distribution, and neurological adaptation [26,27]. The identification of these genetic variants provides insights into the mechanisms underlying athletic performance and highlights the genetic complexity involved in achieving optimal athletic prowess, as illustrated in Table 1.

Despite the valuable insights offered by current research, several important limitations must be acknowledged when interpreting genetic associations with athletic performance. First, many studies are based on relatively small sample sizes, which limits statistical power and increases the likelihood of false-positive findings. Second, most studies are conducted in specific ethnic or geographic populations, raising concerns about the generalizability of results to broader or more diverse athletic cohorts. Third, the human genome is highly complex, and athletic traits are likely shaped by intricate gene–gene and gene–environment interactions that remain poorly understood. These factors collectively highlight the need for larger, multi-ethnic cohort studies and integrative analyses that account for environmental influences such as training load, nutrition, and psychosocial stressors.

When it comes to injury susceptibility, several practical studies have found that genomics plays a significant role. Specific gene variants, such as those in the *MMP* group (matrix metalloproteinase group, rs591058, and rs679620, which are involved in tissue remodeling and repair) and *COL5A1* (rs13946, responsible for collagen synthesis and tendon strength), strongly correlate with increased injury risk among competitive athletes [36,37]. Replicated associations have highlighted polymorphisms like *ACTN3* (rs1815739), *ACAN* (rs1516797), and *VEGFA* (rs2010963) (linked with muscle fiber composition, power generation, cartilage structure, joint function, angiogenesis, and tissue oxygenation) in predisposing athletes to various non-contact injuries [38]. Additional genotypes, including *GDF5*, *AMPD1*, *CO5A1*, and *IGF2* (involved in joint and bone development, energy metabolism in muscle, collagen production for tendon integrity, tissue growth, and repair), have been linked to decreased match participation due to injury risk [39]. Although still in the early research stages, these findings underscore the potential that sports physicians could use genetic data to help them tailor injury prevention strategies for their athletes through a comprehensive understanding of genetic factors.

Research on the role of genetic variations in tissue repair quality is limited, but emerging evidence suggests that variations in collagen genes, such as *COL5A1*, may influence connective tissue integrity, potentially affecting repair quality after injury [40]. Genes involved in muscle regeneration, like *ACTN3*, could modulate recovery capacity from exercise-induced or sports-related muscle damage [41]. Additionally, genetic variations associated with inflammatory responses, cytokine production, and immune function may influence an athlete’s injury recovery efficacy [36,42].

One well-established part of genetic analyses is their role in identifying individuals with inherited diseases that pose serious health risks, such as sudden cardiac death on the field. This condition in athletes primarily arises from genetically influenced cardiac disorders, both structural and electrical. Structural disorders include hypertrophic cardiomyopathy (HCM), the most prevalent cause, accounting for 35% of confirmed cases in the U.S. [41], characterized by mutations in genes like *MYH7*, *MYBPC3*, and *TNNT2* [43]. Primary electrical disorders include long-QT syndrome (LQTS), short-QT syndrome, Brugada syndrome, and catecholaminergic polymorphic ventricular tachycardia. LQTS, the most common electrical disorder causing sudden cardiac death, is often attributed to mutations in the *KCNQ1* gene (LQT1) and *KCNH2* (LQT2) [43]. Sudden non-cardiac death can result from pulmonary, infectious, cerebrovascular, and neurological diseases, all showing genetic associations [43]. These conditions may be exacerbated during high-intensity exercise, emphasizing the need for prevention programs among elite athletes to mitigate sudden cardiac death risk.

Having said all of this, what can a sports physician do to leverage the breadth of scientific knowledge in this field? Medical professionals working with athletes could integrate genomic information into their clinical practice. Today, gathering genomic data is relatively affordable and straightforward, usually through a simple mouth swab. Understanding genetic variants influencing endurance, power, and strength allows for tailored training programs that harness each athlete’s unique predispositions. Identifying genetic markers associated with increased injury susceptibility enables targeted prevention strategies, whereas genetic variations affecting tissue repair quality can inform personalized rehabilitation protocols. Recognizing the genetic basis of inherited diseases, particularly cardiac disorders, underscores the importance of thorough screening and proactive management. By incorporating these genomic insights, medical professionals can optimize athletes’ performance, enhance their overall well-being, and potentially prolong their sports careers.

While genomics in sports medicine offers vast potential benefits, several challenges must be addressed. Ethical concerns regarding privacy, discrimination, and data misuse necessitate clear guidelines. Accurate interpretation of genetic information requires trained professionals or additional education of sports physicians, and educational barriers could hinder implementation. The cost, while generally not expensive, might still be a barrier for teams with tight budgets. And not to forget, in a competition-packed season, time might be lacking for detailed analyses and integration of data into the existing medical history of athletes. However, the benefit of genetic testing, especially when it comes to identifying individuals with a higher risk of cardiac disorders and cardiac death, should outweigh the challenges, not to mention the potential for incremental improvements in performance and recovery.

### 3.2. Pharmacogenomics

PGx offers a transformative approach to understanding how individual genetic variations influence drug response, enabling personalized treatment strategies. Research indicates that over 90% of individuals possess at least one actionable PGx variant, which may predispose them to adverse drug reactions, therapeutic failure, or hypersensitivity [44]. By tailoring drug selection and dosage to a patient’s genetic profile, PGx has the potential to overcome the limitations of conventional trial-and-error prescribing practices, which remain a leading cause of drug-related morbidity and mortality globally [45]. This article examines the application of PGx in sports medicine, focusing on its role in optimizing therapeutic outcomes, enhancing recovery, and improving overall performance in athletes.

The cytochrome P450 (CYP) enzyme system, a central element in drug metabolism, is subject to genetic variation, resulting in different metabolizer phenotypes: poor (PM), intermediate (IM), extensive (normal) (EM), rapid (RM), and ultra-rapid metabolizers (UM). These variations impact the speed and efficiency of drug metabolism, with significant implications for both treatment efficacy and the risk of adverse events. Understanding an athlete’s CYP metabolizer status allows for more precise medication management, ultimately supporting individualized treatment plans.

In clinical practice, genomic data can be easily obtained either through a venous blood draw or a simple, non-invasive mouth swab, allowing physicians to determine an athlete’s metabolizer phenotype. This information enables the personalization of drug therapy based on the person’s metabolic capacity, thereby improving safety, reducing the likelihood of adverse reactions, and optimizing drug efficacy. Such an approach is particularly valuable in the management of elite athletes, where precision in treatment is paramount to ensuring both performance and recovery.

In sports medicine, PGx can enhance both performance and recovery by reducing drug side effects and optimizing treatment efficacy. Genetic variations account for 25–90% of the differences in how individuals respond to medications [46]. Women, in particular, experience 10.2% more adverse drug events than men, due to sex-based differences in drug metabolism, which result in higher drug exposure and an increased risk of side effects [47].

Athletes, compared to non-athletes, tend to use prescription and over-the-counter medications more frequently—especially antibiotics and analgesics—due to the high microbial contamination in sports environments and the gut microbiome changes caused by travel [48,49]. Genetic differences play a significant role in determining drug responses, influencing the occurrence of side effects, toxicity, and treatment effectiveness [50].

As the incidence of sports-related injuries rises, so does the use of analgesics. Injury rates vary across sports, from 2.8% in tennis to 54.2% in soccer [51]. Female soccer players are particularly vulnerable, with a 35% increase in ACL injuries [52]. Non-steroidal anti-inflammatory drugs (NSAIDs) are the most commonly prescribed medications [53]. Of the 8.6 million sports injuries occurring annually, many require surgical intervention and postsurgical opioid pain management [54]. Among athletes, 4.4% to 4.7% use opioids, with usage rates as high as 52% in the National Football League [55]. Contributing risk factors include Caucasian ancestry, participation in contact sports, and undiagnosed concussions [55]. Genetic variants can lead to adverse side effects or insufficient pain relief from analgesics, potentially prolonging recovery and negatively affecting athletic performance.

The Clinical Pharmacogenetics Implementation Consortium (CPIC) and the Dutch Pharmacogenetics Working Group (DPWG) are key organizations that provide evidence-based guidelines for the clinical implementation of PGx, aiming to optimize drug therapy through the use of genetic information. In this paper, we present evidence and highlight specific gene–drug pairs that sports medicine practitioners can integrate into their practice. These guidelines will enable personalized treatments for common conditions encountered in sports medicine, including pain management, infections, cardiovascular health, and mental health disorders. By incorporating PGx into clinical practice, sports medicine professionals can improve treatment outcomes and minimize adverse drug reactions in athletes.

### 3.3. Pain Management

NSAIDs and CYP2C9: Due to interindividual variability in NSAID response, CPIC guidelines recommend 25–50% of the lowest starting dose for CYP2C9 PMs and the lowest starting dose for IMs for drugs like celecoxib, flurbiprofen, ibuprofen, and lornoxicam [56].

Opioids and CYP2D6: Genetic variants of CYP2D6 affect the metabolism of opioids like codeine and tramadol [57]. CPIC recommends alternative opioids for CYP2D6 PMs due to a lack of efficacy and for UMs due to increased side effect risk. Other genes like COMT and OPRM1 modulate pain response but lack specific PGx guidelines.

Muscle Relaxants and CACNA/RYR1: Most oral muscle relaxants lack PGx guidelines. CPIC recommends avoiding desflurane, enflurane, halothane, isoflurane, methoxyflurane, sevoflurane, and succinylcholine in individuals with malignant hyperthermia susceptibility [58].

### 3.4. Cardiovascular Conditions

Athletes are at an elevated risk for cardiovascular conditions such as atrial fibrillation [59] and venous thrombosis, due to factors like intense training, immobilization following injuries, and extended travel [60]. Anticoagulants and antiplatelet agents, commonly used as first-line therapies, demonstrate significant interindividual variability in drug response. In athletes with familial hypercholesterolemia, early intervention may be necessary. Statins, typically the first-line treatment, are associated with an increased risk of myopathy, a condition exacerbated by physical exertion [61]. Beta-blockers and antihypertensives are frequently prescribed for the management of acute cardiac events, hypertension, and tachycardia in athletes.

Anticoagulants and Antiplatelets: Warfarin dosing uses a CPIC algorithm considering age, weight, medications, and genetics (CYP2C9, CYP2C cluster, CYP4F2, VKORC1). For clopidogrel, the FDA, CPIC, and DPWG recommend alternatives for CYP2C19 PMs due to reduced drug activation and higher cardiovascular risks [62].

Anti-lipids: *SLCO1B1* variants (e.g., rs4149056) increase statin-induced myopathy risk. CPIC and DPWG suggest alternative statins or dosing adjustments based on genotype.

Beta-blockers: CYP2D6 genetic variants play a crucial role in determining drug levels and patient response. The U.S. Food and Drug Administration (FDA) advises against using carvedilol and metoprolol in CYP2D6 PMs due to the heightened risk of adverse effects such as bradycardia and dizziness. For metoprolol, the DPWG recommends reducing the dose to 25–50% of the standard or using a slower titration approach in PMs, while UMs may require up to 2.5 times the standard dose to achieve therapeutic efficacy. Additionally, variants in the *ADRB1* and *ADRB2* genes influence the risk of drug-induced hypotension and treatment response with beta-blockers like bisoprolol [63].

### 3.5. Infectious Disease

Athletes risk infectious diseases due to close contact, intense training, travel, and pathogen exposure. Some need drug prophylaxis for conditions like bacterial endocarditis, hepatitis, and HIV. Antibiotics, antifungal, and antiviral medications with PGx biomarkers offer safer, more effective therapy.

Antibiotics: The CPIC guidelines recommend avoiding aminoglycosides (amikacin, gentamicin, kanamycin, paromomycin, plazomicin, streptomycin, tobramycin) in individuals with high-risk *MT-RNR1* variants (1095T > C, 1494C > T, 1555A > G) due to ototoxicity risk [64].

Antifungals: The CPIC and DPWG recommend alternative antifungals for CYP2C19 PM, RM, and UM, avoiding voriconazole [65].

Antivirals:

- Abacavir: Avoid in individuals with HLA-B*57:01 variant due to hypersensitivity risk. The FDA and EMA recommend testing before use in HIV patients [66].
- Atazanavir: CPIC warns *UGT1A1* PMs of increased jaundice risk, potentially leading to non-adherence. UGT1A1 PMs often have the Gilbert syndrome phenotype [67].
- Efavirenz: Reduce dose to 200–400 mg/day for CYP2B6 PMs due to side effect risk; start at 400 mg/day for IMs [68].

### 3.6. Psychotropics: Antidepressants, Anti-Seizure Medications

Antidepressants (SSRIs, SNRIs, Tricyclics): Genetic variations in CYP2B6, CYP2C19, and CYP2D6 significantly affect plasma levels and outcomes. CPIC guidelines recommend using antidepressants not metabolized by CYP2C19 for UMs and PMs, with dose adjustments if necessary. CYP2D6 PMs may need lower doses or alternatives due to side effects, while UMs may need higher doses or alternatives for efficacy. For tricyclics like amitriptyline and clomipramine, CPIC suggests alternative drugs for CYP2D6 UMs or PMs and CYP2C19 UMs, RMs, and PMs. If necessary, a 50% dose reduction is recommended for CYP2D6 or CYP2C19 PMs and a 25% reduction for CYP2D6 IMs [69,70].

Anti-seizure Medications (Carbamazepine, Phenytoin/Fosphenytoin, Oxcarbazepine): CPIC guidelines recommend alternative drugs for carbamazepine-naive patients with HLA-B*15:02 or HLA-A*31:01 alleles due to risks of SJS, TEN, DRESS, and MPE. Phenytoin and fosphenytoin are contraindicated for individuals with HLA-B*15:02 due to SJS and TEN risks. Patients with CYP2C9 PMs phenotype or activity score of 1 may need reduced doses. For oxcarbazepine-naive patients with HLA-B*15:02, CPIC recommends alternative drugs due to SJS and TEN risks [71,72].

### 3.7. Multi-Omics

**Figure 1:**
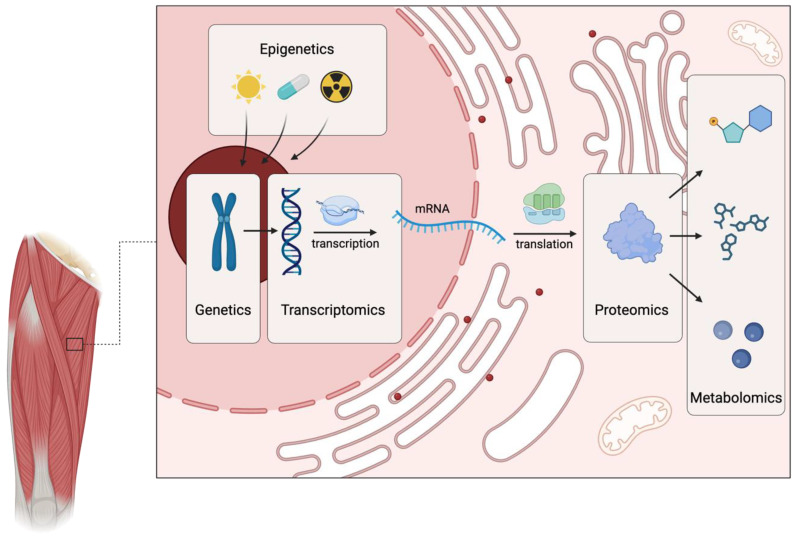
Components of multi-omics.

Multi-omics integrates data from multiple biomolecular domains—genomics, epigenomics, transcriptomics, proteomics, and metabolomics (Figure 1) [5]. Genomics identifies gene variants impacting athletic traits such as muscle fiber-type composition and injury susceptibility. Epigenomics explores reversible modifications that modulate gene expression, with evidence suggesting exercise-induced epigenetic remodeling in skeletal muscle mediates training adaptations [73]. Transcriptomics profiles RNA molecules, revealing distinct signatures in skeletal muscle in response to various exercise regimens [74]. Non-coding RNAs, like microRNAs, serve as potential, albeit still preliminary, biomarkers of training status and physical stress [75]. Proteomics characterizes the entire protein complement, including abundance and modifications. Studies have demonstrated that exercise significantly remodels the muscle proteome, potentially influencing strength, endurance, and recovery capacity [76,77]. Lastly, metabolomics profiles small-molecule metabolites to identify exercise-induced alterations in biofluids associated with energy utilization, oxidative stress, and fatigue. These changes may indicate potential biomarkers for training status and recovery [78], offering valuable insights into an athlete’s physiological state.

Implementing multi-omics, or at least several aspects of it, into regular sports medicine evaluations may help in creating a comprehensive systems-level perspective of biological processes influencing the athlete’s physiology and pathophysiology, offering potential insights into their performance, injury predisposition, and recovery dynamics in sports medicine. While this field in the context of sports medicine is still in its infancy, familiarizing sports physicians with the latest advancements is a valuable step in the right direction.

Despite its early days in sports, studies have found potentially meaningful discoveries that may enhance the overall health and care of athletes. A study that included a proteogenomic analysis identified a plasma proteomic signature enriched for proteins involved in cellular stress response pathways, which correlated with enhanced cardiovascular fitness parameters such as maximal oxygen uptake (VO_2_max) [79]. Epigenome-wide association studies have linked specific DNA methylation patterns in genes regulating skeletal muscle development and function to elite athlete status [80].

For injury risk stratification and prevention, an integrative multi-omics approach identified a panel of circulating metabolites, proteins, and miRNAs that could discriminate between individuals with and without Achilles tendinopathy, a common overuse injury in athletes [81]. This molecular signature could potentially serve as a non-invasive screening tool for early detection in at-risk athletes. Longitudinal multi-omics profiling has revealed exercise-induced alterations in the muscle transcriptome and metabolome that precede clinical manifestations of exertional rhabdomyolysis [82], enabling timely monitoring and mitigation strategies to prevent acute injury episodes.

In recovery and rehabilitation, multi-omics data can help better understand the biological processes underlying tissue repair and remodeling. A recent proteomics study identified distinct temporal patterns of skeletal muscle protein expression following acute exercise, with an initial upregulation of proteins involved in inflammation and muscle repair, followed by a later increase in proteins mediating metabolic adaptation [83]. Such findings provide a molecular basis for developing strategic nutritional or therapeutic interventions synergistic with the distinct phases of the muscle recovery process, potentially accelerating healing and restoring optimal function.

Moreover, multi-omics approaches can help with other aspects of an athlete’s overall well-being, and one of the most important ones is sleep quality and mental health. Sleep plays a crucial role in athletic performance, recovery, and injury prevention. Metabolomic analyses have revealed associations between specific metabolite signatures and sleep quality in athletes, suggesting potential links between metabolic homeostasis and recovery processes [84]. For instance, a study found that poor sleep quality in elite athletes was associated with altered levels of metabolites involved in energy metabolism, such as glucose, fatty acids, and amino acids [13]. Additionally, proteomic studies have identified changes in the expression of proteins related to circadian rhythms and stress response in athletes with sleep disturbances [85].

While the integration of multi-omics data holds significant potential for enhancing sports medicine practices, as with other novel approaches, several key challenges should be noted. Robust computational pipelines and analytical approaches are needed to effectively integrate and interpret the vast, heterogeneous datasets, and this can be challenging to interpret for physicians without additional education. While the standardization of experimental protocols is crucial for ensuring data reproducibility and enabling meaningful comparisons across studies, comparing results from athletes from different sports might be challenging due to the different physical requirements for each sport. Then, translating multi-omics research findings into actionable clinical decision support tools is a significant bottleneck, especially since these studies are in their infancy in sports medicine. Logistical and ethical considerations, such as managing large-scale datasets, ensuring data privacy, and navigating regulatory frameworks, must also be carefully addressed. Despite these challenges, the potential benefits of multi-omics for optimizing athlete health, performance, and longevity are promising, hence why continued research efforts and overcoming existing barriers could be worthwhile in the long-term scheme.

### 3.8. Digital Health

The digital health umbrella represents one of the most promising innovations in sports medicine. It encompasses advanced devices and technologies like wearable sensors and wearable devices that can help in continuous and remote monitoring of athletes [86]. These devices capture physiological metrics, real-time training loads, recovery indicators, sleep quality, and mental health factors, enabling a highly individualized approach to each and every athlete. It also encompasses telemedicine, allowing real-time, remote consultations and continuous monitoring [87]. This highly individualized approach leveraging digital health can yield customized training regimens, nutrition plans, injury recovery management, and targeted injury prevention strategies [88]. Furthermore, data collection is a crucial component of digital health, forming the foundation for future research into the health optimization of athletes.

### 3.9. Wearable Sensors and Devices

Wearable devices are a collective term for various devices used as tools for monitoring an individual’s physical condition or enhancing it. These devices can be categorized into several groups, such as smartwatches, smart jewelry, smart clothing, augmented reality glasses, or various specialized medical devices. Devices from these categories are capable of tracking different physiological parameters and are highly accepted as great tools in treatment and the prevention of potential pathological conditions [89]. These devices also collect information on multiple such data points and, based on their analysis, draw conclusions about an individual’s overall state, providing recommendations related to sleep, rest, stress levels, etc. These devices are particularly beneficial for athletes as they not only allow for the direct acquisition of extremely useful data but also contribute to improved performance, injury prevention, and, of course, enhanced health. Some of the blood parameters whose data can be tracked using this technology include blood and muscle oxygen saturation, using, for example, luminescent oxygen sensors for transcutaneous oxygen monitoring [90] or optical wearable devices provided by near-infrared spectroscopy technology. This technology measures the changes in the amount of oxygen which is bound to chromophores (e.g., hemoglobin, myoglobin, and cytochrome oxidase) at the target tissue level [91]. Also, it is possible to non-invasively monitor blood glucose [92]. Knowledge of these values, in addition to their obvious potential for optimizing nutrition, also clearly contributes to a better understanding of an athlete’s physical fitness, specifically how much of the effort is determined aerobically or anaerobically. Additionally, it is possible to obtain knowledge of more complex medical data such as electrocardiogram and electromyography using multimodal low profile wearable data acquisition device [93], which provides insights into muscle activation patterns, fatigue, and efficiency, but can also contribute to the prevention of life-threatening conditions such as sudden cardiac death or other potential cardiac pathologies. Beyond these parameters, it is possible to collect a whole host of others, such as hydration and sweat analysis, body temperature, respiratory rate, heart rate, and heart rate variability [93,94]. This continuous monitoring helps detect serious pathologies as well as dehydration-related performance decreases and health risks. Wearable devices also facilitate personalized nutrition strategies tailored to each athlete’s unique metabolic profile and training demands. By extrapolating individual values and analyzing them in relation to each other, information on the amount of stress, post-competition recovery, or pre-game fitness can also be obtained. Finally, by precisely measuring the individual stages of sleep, sleep latency, patterns, duration, and quality, wearable devices such as Whoop [95] or Oura ring [96] enable the monitoring and improvement of sleep, which is paramount for optimal recovery and performance [97,98].

As discussed earlier, wearable devices and tracking systems capture comprehensive data on player performance metrics. These data enable coaches to make decisions on player selection, substitutions, and training program design to customize it, addressing individual weaknesses while capitalizing on strengths. Many studies are showing the real-world benefits of wearable devices. For example, the acute-to-chronic workload ratio (ACWR) is a metric that can be measured by wearables, and it is very useful to assess an athlete’s training load and potential risk of injury or overtraining. The ACWR is calculated by dividing the acute workload by the chronic workload, and a higher value indicates a sudden increase in training load compared to the athlete’s usual level [99]. The ACWR has proven crucial in injury risk assessment, with athletes exhibiting an ACWR greater than 1.7 being almost fivefold more likely to sustain a contact injury [100]. In professional men’s soccer, where injury incidence is 8.7 per 1000 h of exposure [101], it is obvious just from this one example how wearable devices play a vital role in lowering injury risk. Another study performed among eight National Collegiate Athletic Association Division I teams across different sports also proved that athletes who utilized wearable devices and acted upon the acquired data had a 60% decrease in injuries [102]. The same study also showed a decrease in resting heart rate and heart rate variability, an improvement in sleep time and quality, and an improvement in key biomarkers of stress.

A study demonstrated that digital health systems integrating wearable data, body composition analysis, and artificial intelligence algorithms could accurately predict individual energy requirements within a narrow margin of error [103]. This level of precision enables athletes to optimize their caloric and macronutrient intake, supporting muscle recovery, replenishing energy stores, and maintaining overall health.

Despite the numerous benefits of digital health technologies in sports, there are several limitations and challenges to consider. Data privacy and security concerns are paramount, as athletes’ sensitive health information is collected and transmitted. There is also the risk of over-reliance on technology, potentially leading to a neglect of subjective athlete feedback and intuitive coaching. The accuracy and reliability of some wearable devices may be questionable, especially in high-intensity or water-based sports. Additionally, the cost of implementing and maintaining these technologies can be prohibitive for many teams or individual athletes. There is also a learning curve associated with interpreting the vast amount of data generated, which may overwhelm coaches and athletes without proper training. Furthermore, the rapid pace of technological advancement can quickly render expensive equipment obsolete. Lastly, there is the challenge of integrating data from various devices and platforms, as a lack of standardization can lead to inconsistencies in data interpretation and application. These limitations underscore the need for a balanced approach that combines technological insights with traditional sports science and coaching methodologies.

Overall, the data demonstrate that the application of digital health technologies enables the optimization of numerous aspects of an athlete’s overall life. The primary benefit derived from these devices, achieved through data collection and analysis, is their effective integration, primarily in the form of personalized training, nutrition, and sleep. Such a regime and approach to sports, or rather to the individual athlete, allows for more efficient progress and better performance while simultaneously reducing the risk of injury, preventing overtraining, etc. The integration of digital health tools supports psychological well-being by reducing stress, improving sleep quality, and promoting mental health. Tailored macronutrient intake, aligned with individual athlete needs, further enhances both professional performance and overall health, which is another one of the many benefits of incorporating cutting-edge technologies in sports science.

### 3.10. Telemedicine

Telemedicine, the delivery of healthcare services remotely through information and communication technologies, has emerged as an important part of sports medicine. This digital health solution has gained substantial traction in recent years, revolutionizing how sports medicine professionals interact with athletes and their support teams. By leveraging advanced telecommunications infrastructure, telemedicine in sports aims to overcome geographical barriers, enhance accessibility to specialized care, and streamline healthcare delivery for athletes at all levels. When implemented effectively, telemedicine has the potential to significantly improve the triad of the following healthcare goals: accessibility, cost-effectiveness, and quality of care [104]. It offers a paradigm shift in clinical practice, allowing sports medicine providers to allocate more time and resources to direct athlete care by reducing administrative burdens and eliminating unnecessary in-person appointments. Moreover, telemedicine serves as a powerful tool for breaking down information exchange barriers, fostering collaborations among sports healthcare professionals, coaches, and athletes that ultimately lead to more comprehensive and effective care strategies.

A strong point of telemedicine is that it allows athletes to receive remote consultations with specialists, greatly facilitating preliminary assessments [105]. This is particularly valuable, as data indicates that only less than 3% of sports- and recreation-related injury episodes resulted in hospitalization [54]. Telemedicine has been well-received, with most patients expressing satisfaction with virtual consultations, which demonstrated shorter wait and visit times compared to inpatient appointments [106].

Moreover, there is a wide span of pathologies in which telemedicine can be applied. Some of them are more specific, like cystic fibrosis or retinal pathologies, while some include the whole field, such as dermatology or even vascular surgery.

While telemedicine offers numerous benefits, it also faces several challenges and limitations in the context of sports medicine. One significant concern is the potential for missed diagnoses or misinterpretations due to the lack of hands-on physical examinations. Certain injuries or conditions may require in-person assessments for accurate diagnosis and treatment planning. Additionally, there are concerns about data security and patient privacy, especially when transmitting sensitive medical information over digital platforms. The digital divide can also pose a barrier, as not all athletes may have access to reliable internet connections or necessary devices, potentially exacerbating healthcare disparities. Moreover, the adoption of telemedicine requires significant investment in technology infrastructure and training for healthcare providers, which can be a hurdle for some organizations. Lastly, there are ongoing discussions about licensure and reimbursement policies for telemedicine services, which vary across different regions and can impact their widespread implementation. Despite these challenges, as technology continues to advance and regulatory frameworks evolve, telemedicine is likely to play an increasingly important role in providing accessible, efficient, and personalized care for athletes at all levels of competition.

### 3.11. Future Perspectives on Enhancing Athlete Well-Being and Performance Through Digital Health

As sports medicine continues to evolve, emerging technologies and artificial intelligence (AI) present exciting possibilities for enhancing athlete care, performance optimization, and research methodologies. While still in early stages of development and implementation, these innovations have the potential to transform various aspects of sports medicine practice.

AI shows promise in enhancing data analysis for sports medicine research, offering new capabilities in processing complex datasets. Machine learning algorithms may help identify patterns in large datasets that are challenging for human researchers to detect, potentially providing new perspectives on injury prevention, performance, and recovery. For example, AI could assist in analyzing diverse athlete data—such as biomechanics, physiological markers, and training loads—to explore potential early indicators of overtraining or injury risk. Natural language processing techniques might aid in reviewing and summarizing findings from numerous research papers, potentially expediting literature reviews in the field.

One area of growing interest is the use of integrated technology and real-time data to optimize athlete preparation. Wearable sensors capturing parameters such as core temperature, muscle activation, and heart rate variability could enable more individualized warm-up protocols. While further research is needed, such systems may improve readiness and reduce the risk of overexertion. Integrated approaches like these have been suggested to enhance preparation, training, and recovery in field-based team sports [107].

Another exciting possibility is tracking traditionally challenging-to-measure factors, such as the impact of motivational speeches on athletes’ physiological and emotional states. By leveraging sensors to monitor parameters like heart rate, skin conductance, and brain activity, coaches and sports psychologists could objectively assess the effectiveness of different motivational approaches. These data could then be used to craft more impactful speeches that resonate best with individual athletes, enabling strategic integration into pre-competition routines.

Leveraging AI could help to enhance mental well-being and psychological resilience in athletes [108]. AI-driven emotion recognition systems, analyzing physiological markers and facial expressions, could provide insights into an athlete’s emotional state. This would enable sports psychologists to implement personalized interventions, such as guided meditation or visualization techniques, to cultivate a positive mindset and foster emotional resilience.

While the potential applications of AI and advanced technologies in sports medicine are promising, it is crucial to approach their implementation with careful consideration. As these technologies continue to develop, scientific validation will be necessary to ensure their effectiveness, reliability, and safety in real-world sports medicine settings. Ethical considerations, including data privacy, equity of access, and the balance between technological assistance and human expertise, will need to be thoroughly addressed. Moreover, the integration of these technologies into existing sports medicine practices will require interdisciplinary collaboration among medical professionals, data scientists, engineers, and ethicists. As the field moves forward, maintaining a patient-centered approach and preserving the fundamental human elements of healthcare will be paramount.

### 3.12. Proposed Framework and Conclusions

The integration and utilization of genomics, PGx, digital health solutions, and multi-omics data represent a promising opportunity for athlete care in sports medicine. While many are still in their early periods of development in sports medicine, these interdisciplinary approaches could provide a high level of personalization in the future, impacting athletic performance, competitive longevity, injury risk, and recovery, as well as overall athlete well-being. Today, there are multiple ways to leverage these technologies, such as using wearable devices to track sleep, fatigue, recovery, and overall well-being. Utilizing PGx could guide more targeted treatments and higher effectiveness when using medications in athletes. Genetic tests to identify athletes who may be at higher risk of cardiac abnormalities or other medical conditions could be one of the main ways of minimizing the risk of fatal outcomes, such as sudden cardiac death. This integrated approach using the aforementioned solutions that are part of the next generation precision sports medicine may benefit athletes throughout their professional careers and have profound implications for their overall health, quality of life, and longevity beyond their competitive years. As science and technology continue to evolve, offering tangible opportunities for optimizing athlete care and performance may become an everyday part of sports medicine for physicians, regardless of the sport.

**Figure 2:**
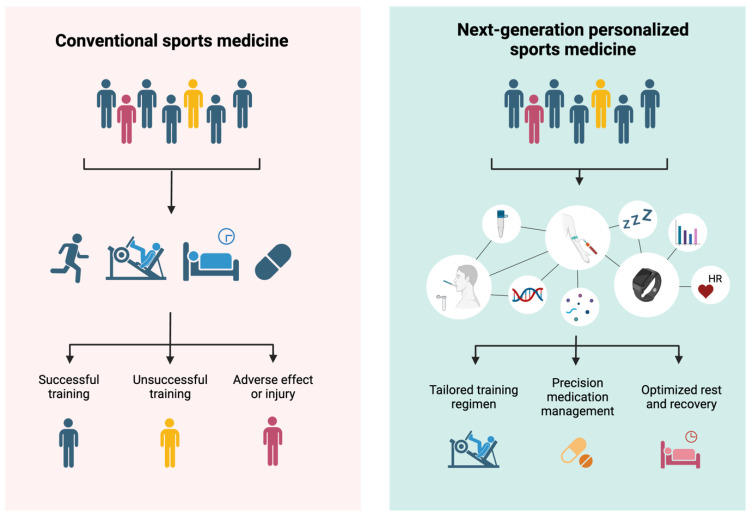
Framework of personalized sports medicine.

To aid physicians in understanding current and future scientific and technological advancements in sports medicine, as well as their tangible benefits for athletes’ performance and recovery, we provided a framework in Figure 2, which highlights the tests and technologies that form the foundation of next-generation precision sports medicine and are ready for practical application. In brief, we recommend a comprehensive approach to optimize athlete care and performance.

The first step could involve broad assessments using wearable devices to monitor real-time physiological parameters such as heart rate variability, sleep quality, muscle oxygenation, and hydration. These data will help track training loads, recovery, and overall athlete well-being. Simultaneously, at the beginning of an athlete’s career, genetic testing should be performed to design personalized training regimens and preventive strategies, with a particular focus on cardiac health, injury predisposition, and performance-related genetic traits. Before starting any medication regimen, PGx testing should be conducted to tailor medication management—particularly for commonly used drugs such as NSAIDs, muscle relaxants, or recovery supplements—ensuring optimal therapeutic responses and minimizing adverse effects.

Throughout the competitive season, continuous physiological monitoring via wearable technology should be used to assess training loads, recovery status, and overall health. These data should be regularly analyzed to adjust training protocols and identify early signs of overtraining. During off-seasons or recovery periods, a more in-depth analysis using multi-omics profiling (e.g., proteomics and metabolomics) can provide deeper insights into individual physiology, enabling even more targeted interventions.

Based on these assessments, highly individualized interventions can then be crafted. For instance, if wearable data signals increased injury risk or overtraining, training loads can be adjusted accordingly to prevent injury. PGx data will allow for precise drug dosing, reducing side effects and speeding up recovery. If more granular insights are required, multi-omics profiling can be used to tailor nutrition plans, optimize muscle recovery, and enhance overall performance. This multi-layered, data-driven approach ensures that physicians can deliver highly personalized care throughout an athlete’s career, boosting both performance and long-term health outcomes.

While the potential benefits of integrating advanced technologies into sports medicine are significant, numerous challenges must be addressed. The complexity of managing and interpreting vast, heterogeneous datasets from various sources presents a major hurdle, as does the translation of research findings into practical clinical tools. Ethical considerations surrounding genetic data use, along with concerns about data privacy, security, and interoperability of digital health technologies, pose significant obstacles. The high costs associated with some of the novel technologies may limit accessibility, potentially exacerbating inequalities in athlete care. Additionally, there is a pressing need for rigorous validation of newer wearable sensors and remote monitoring devices to ensure their reliability in sports-specific contexts. Regulatory hurdles, resistance to change from some practitioners, and the unknown long-term effects of relying heavily on these technologies complicate their integration. Overcoming these challenges will require collaborative efforts from multiple disciplines, as well as the development of comprehensive frameworks for responsible and ethical implementation in athlete care.

In conclusion, the future of sports medicine lies in providing highly personalized care for athletes, which can in a significant part be achieved with the integration of genomics, PGx, digital health solutions, and multi-omics data. While these technologies offer promising opportunities to enhance performance, prevent injuries, and promote overall well-being, their implementation is not without challenges. As the field evolves, it is important for the sports medicine community to embrace these next-generation approaches responsibly, balancing innovation with ethical considerations and athlete welfare. By doing so, we can not only elevate athletic performance but also contribute to their long-term health and quality of life beyond their competitive years. The journey toward this integrated, personalized approach in sports medicine has begun, and its potential to transform athlete care is both exciting and profound.

## Figures and Tables

**Table 1:** Genes associated with athletic performance or injury risk.

Genetic Marker	Associated Role
*AMPD1* rs17602729 C	favorable for sprint and power performance [28]
*CDKN1A* rs236448 A	associated with a greater proportion of fast-twitch muscle fibers and a predisposition to power sports [22] associated with longer durations of exercise % [22]
*CKM* rs8111989	muscle-specific creatine kinase [29] linked to injury predisposition [29]
*MYBPC3* rs1052373 G	associated with increased VO_2_max and elevated levels of the testosterone precursor androstenediol, which potentially contributes to the superior performance of endurance athletes [23]
*ACE* rs4646994 D	associated with higher levels of angiotensin-converting enzyme, a greater proportion of fast-twitch muscle fibers, and superior performance in power and sprint sports [29]
*NFIA-AS2* rs1572312 C	associated with endurance [30] high VO_2_max in three subgroups of athletes (male and female middle and short endurance athletes; female long endurance athletes) [30]
*PPARA* rs4253778 G	exercise-induced LV growth [31] improved aerobic performance [31]
*PPARGC1A* rs8192678 G	enhanced mitochondrial efficiency, higher aerobic capacity, and a greater proportion of fatigue-resistant type I muscle fibers [32]
*ACTN3* rs1815739 C	associated with muscle fiber composition, may confer some advantage in power performance events [33]
*CPNE5* rs3213537 G	associated with an increased proportion of fast-twitch muscle fibers and better sprint times compared to endurance athletes and controls [24]
*GALNTL6* rs558129 T	favorable for anaerobic performance and strength athletes better short-distance swimming athlete status [25]
*NOS3* rs2070744 T	higher prevalence among elite power athletes compared to endurance athletes and non-athletic controls [34]
*AR* ≥ 21 CAG repeats	associated with higher fat-free mass and serum testosterone levels [26,27]
*COL5A1* rs12722 T	increased risk of tendon and ligament injuries (e.g., Achilles tendinopathy, ACL rupture) [35]

## Data Availability

The data for this narrative review are available in the referenced articles.

## Abbreviations

PGx	Pharmacogenomics
DH	Digital Health
SNP	Single Nucleotide Polymorphism
CYP	Cytochrome P450
PM	Poor Metabolizer
IM	Intermediate Metabolizer
EM	Extensive (Normal) Metabolizer
RM	Rapid Metabolizer
UM	Ultra-Rapid Metabolizer
CPIC	Clinical Pharmacogenetics Implementation Consortium
DPWG	Dutch Pharmacogenetics Working Group
NSAIDs	Non-Steroidal Anti-Inflammatory Drugs
SSRIs	Selective Serotonin Reuptake Inhibitors
SNRIs	Serotonin-Norepinephrine Reuptake Inhibitors
TCA	Tricyclic Antidepressant
ECG	Electrocardiogram
EMG	Electromyography
AR	Augmented Reality
VO_2_max	Maximal Oxygen Uptake
